# A taxonomic revision of the *Neoserica* (sensu lato) *pilosula* group (Coleoptera, Scarabaeidae, Sericini)

**DOI:** 10.3897/zookeys.440.8126

**Published:** 2014-09-15

**Authors:** Wan-Gang Liu, Silvia Fabrizi, Ming Bai, Xing-Ke Yang, Dirk Ahrens

**Affiliations:** 1 Key Laboratory of Zoological Systematics and Evolution, Institute of Zoology, Chinese Academy of Sciences, Box 92, No. 1, Beichen West Road, Chaoyang District, Beijing, 100101, P.R. China; 2 University of Chinese Academy of Sciences, Yuquan Road, Shijingshan, Beijing, 100039, P.R. China; 3 Zoologisches Forschungsmuseum A. Koenig, Adenauerallee 160, 53113 Bonn, Germany

**Keywords:** Beetles, chafers, *Neoserica*, China, new species

## Abstract

Nine new species of the *Neoserica* (sensu lato) *pilosula* Moser, 1915, group are described from China: *Neoserica curvipenis* **sp. n.**, *N. emeishanensis* **sp. n.**, *N. lincangensis* **sp. n.**, *N. ludingensis* **sp. n.**, *N. lushuiana* **sp. n.**, *N. rangshuiensis* **sp. n.**, *N. shennongjiaensis* **sp. n.**, *N. tianeana* **sp. n.**, and *N. weibaoshanica* **sp. n.** The lectotype of *Neoserica pilosula* Moser, 1915, is designated. Habitus and male genitalia are illustrated, a key to the species of the group and a map of species distribution are given.

## Introduction

*Neoserica* Brenske, 1897 is one of the most species-rich groups of Sericini. It comprises nearly 200 taxa. Since the designation of the type species of *Neoserica* (Pope 1960) and the redefinition of the genus based on a first revision of close allies of the type species (Ahrens 2003), many species are so far grouped under *Neoserica* being not directly related to *Neoserica* sensu stricto (Ahrens 2003). We preliminarily consider them in *Neoserica* sensu lato (e.g. Ahrens 2004), a collective group that was found to be neither monophyletic (Ahrens and Vogler 2008) nor related to *Neoserica* sensu stricto (Ahrens 2003). The current study continues a series of the taxonomic revisions of *Neoserica* species groups (Ahrens et al. 2014a, Ahrens et al. 2014b, Ahrens et al. in press) based on which hopefully their relationship and their right classification can be subsequently established.

In the present paper we explore the taxonomy of the representatives related to *Neoserica pilosula* Moser, 1915, originally described from Yunnan (China). According to our present knowledge, the species group is restricted to the mountain areas of Southwest China. The species of this group are characterised by a bidentate protibia, an antennal club composed of four antennomeres in both sexes, a short labrum that bears a transverse rim of very dense, short and robust setae, and by a densely setose dorsal surface of the body. The *Neoserica pilosula* group shares the transverse rim of setae on labrum with most species of the *Neoserica* (s.l.) *lubrica* group (Ahrens 2004). The species of the latter group, however, have a glabrous dorsal surface and an antennal club composed of three antennomeres in females. Here, nine new species are described, all originating from Southwest China.

## Material and methods

The terminology and methods used for measurements, specimen dissection and genital preparation follow Ahrens (2004). Data from specimens examined are cited in the text with original label contents given in quotation marks verbatim, multiple labels are separated by a “/”. Descriptions, if not otherwise stated, are based on the holotype specimen. Male genitalia were glued to a small pointed card and photographed in both lateral and dorsal view using a stereomicroscope Leica M125 with a Leica DC420C digital camera. A number of single images were combined in order to obtain an entirely focused image using the automontage software as implemented in Leica Application Suite (V3.3.0). The resulting images were subsequently digitally edited to eliminate background using Artweaver software. Based on the geographical coordinates obtained from the labels and Google map (https://www.google.de/maps/), the distribution map was generated using Q-GIS 2.0.1 and Adobe Photoshop CS4 software.

Type specimens and additional material examined are deposited in the following institutions:

CPPB Collection P. Pacholátko, Brno, Czech Republic;

HBUM Museum of Hebei University, Baoding (Hebei Prov.), China;

IZAS Institute of Zoology, Chinese Academy of Sciences, Beijing, China;

NMPC National Museum Prague (Natural History), Czech Republic;

ZFMK Zoologisches Forschungsmuseum A. Koenig, Bonn, Germany;

ZMHB Museum für Naturkunde, Berlin, Germany.

### Key to species groups of *Neoserica* (sensu lato)

**Table:** 

1	Hypomeron not carinate	*Tetraserica* Ahrens, 2004
1’	Hypomeron carinate	2
2	Antennal club in female composed of 3 antennomeres	*Neoserica* (s.l.) *vulpes* group, *Neoserica* (s.l.) *calva* group, *Neoserica* (s.l.) *lubrica* group, *Anomalophylla* Reitter, 1887, *Gynaecoserica* Brenske, 1896, *Leuroserica* Arrow, 1946, *Sericania* Motschulsky, 1860, *Calloserica* Brenske, 1894, *Lasioserica* Brenske, 1896, *Gastroserica* Brenske, 1897, *Neoserica* (s.str.) Brenske, 1894, *Trioserica* Moser, 1922, *Microserica* Brenske, 1894, *Oxyserica* Brenske, 1900, other *Neoserica* (s.l.)
2’	Antennal club in female composed of more than 3 antennomeres	3
3	Labrum without a transverse rim of very dense, short and robust setae	4
3’	Labrum short, with a transverse rim of very dense, short and robust setae. Dorsal surface densely setose	*Neoserica* (s.l.) *pilosula* group
4	Metatibia slender and long	5
4’	Metatibia short and wide	*Neoserica* (s.l.) *uniformis* group & *Neoserica* (s.l.) *multifoliata* group (from Indochina)
5	Antennal club of males with 7 antennomeres	6
5’	Antennal club of males with 7, 6 or less antennomeres	7
6	Metafemur with a continuously serrated line adjacent to the anterior margin of metafemur. Protibia more or less distinctly tridentate	*Neoserica* (s.l.) *septemlamellata* group
6’	Metafemur without a continuously serrated line adjacent to the anterior margin of metafemur. Protibia always distinctly bidentate	*Nepaloserica* Frey, 1965
7	Basis of labroclypeus dull. Antennal club of males with 6 antennomeres	8
7’	Antennal club of males with 5 or 4 antennomeres	9
8	Angle between basis of hypomeron and that of pronotum strongly rounded, angle of surfaces of hypomeron and pronotum basally blunt. Hypomeron basally strongly produced ventrally and transversely sulcate	*Lepidoserica* Nikolaev, 1979
8’	Angle between basis of hypomeron and that of pronotum sharp, angle of surfaces of hypomeron and pronotum sharp. Hypomeron basally not produced ventrally and not sulcate	*Neoserica* (s.l.) *abnormis* group
9	Body surface strongly shiny. Body small (5.7–6.6 mm)	*Neoserica* (s.l.) *speciosa* group
9’	Body surface dull. Body larger (8 mm)	*Chrysoserica* Brenske, 1897

### Key to species of *Neoserica* (s.l.) *pilosula* group (♂ ♂)

**Table:** 

1	Antennal club as long as remaining antennomeres combined	2
1’	Antennal club 1.2 times as long as remaining antennomeres combined	6
2	Eyes smaller: ratio diameter/interocular distance ~ 0.6	*Neoserica ludingensis* sp. n.
2’	Eyes larger; ratio diameter/interocular distance > 0.7	3
3	Metatibia shorter and wider: ratio metatibial width/length < 1/2.9	4
3’	Metatibia longer and narrower: ratio metatibial width/length > 1/3.2	*Neoserica curvipenis* sp. n.
4	Right paramere subequal in length to left or longer	5
4’	Right paramere spherical, much shorter than left	*Neoserica pilosula* Moser
5	Right paramere longer than left	*Neoserica lincangensis* sp. n.
5’	Right paramere subequal in length to left	*Neoserica tianeana* sp. n.
6	Right paramere spherical, much shorter than left. Median apical process between parameres trifid	*Neoserica weibaoshanica* sp. n.
6’	Right paramere long, subequal in length to left or longer	7
7	Right paramere in dorsal view straight	8
7’	Right paramere in dorsal view strongly curved externally	9
8	Right paramere with a filiform spine internally at middle. Left paramere more abruptly narrowed towards apex	*Neoserica shennongjiaensis* sp. n.
8’	Right paramere without a filiform spine internally. Left paramere evenly narrowed towards apex	*Neoserica rangshuiensis* sp. n.
9	Right paramere distinctly longer than width of phallobase at apex	*Neoserica emeishanensis* sp. n.
9’	Right paramere as longer as width of phallobase at apex	*Neoserica lushuiana* sp. n.

## Systematics

### Neoserica (s.l.) pilosula

Taxon classificationAnimaliaColeopteraScarabaeidae

Moser, 1915

Figs 1A–D , 5

Neoserica pilosula Moser, 1915: 377.

#### Type material examined.

Lectotype (here designated) @ “Yünnan China/ Neoserica pilosula Type Mos./ pilosula Mos.” (ZMHB).

#### Additional material examined.

1 ♂ “China C-Yunnan 60km SE Kunming Shilin (Stone forest) lgt. D. Král 3.–4.VII.90” (NMPC), 3 ♂♂, 1 ♀ “Mts. Junzishan, Shizong, Yunnan, 14.VII.2006, leg. Mao Benyong etc.” (HBUM).

#### Redescription of lectotype.

**Figure 1.**
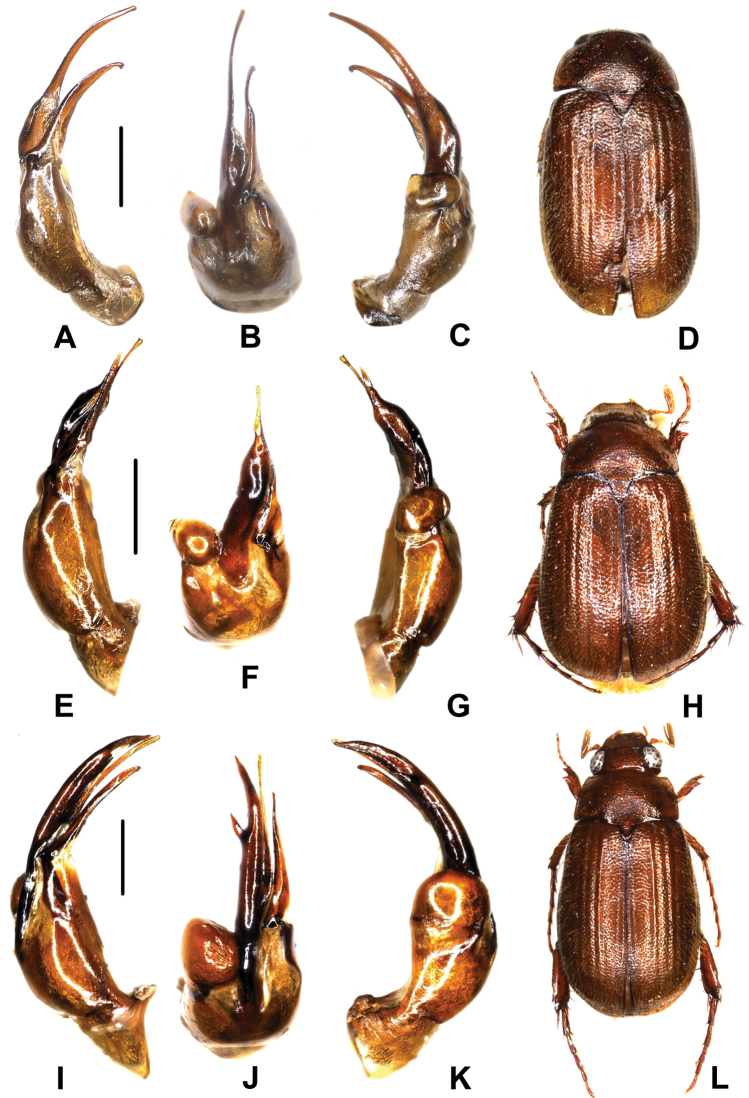
**A–D** *Neoserica pilosula* Moser, 1915 (Lectotype) **E–H** *Neoserica ludingensis* sp. n. (holotype) **I–L** *Neoserica weibaoshanica* sp. n. (holotype). **A, E, I** aedeagus, left side lateral view **C, G, K** aedeagus, right side lateral view **B, F, J** parameres, dorsal view **D, H, L** habitus. Scale: 0.5 mm, habitus not to scale.

Body length: 7.5 mm, length of elytra: 5.7 mm, width: 4.2 mm. Body oblong, reddish brown, antennal club yellowish brown, dorsal surface shiny, densely covered with fine, semi-erect setae (Fig. 1D).

Labroclypeus subtrapezoidal, widest at base; lateral margins weakly convex and moderately convergent towards moderately rounded anterior angles; anterior margin shallowly sinuate medially; margins moderately reflexed; surface moderately elevated medially, coarsely and densely punctate, densely setose. Frontoclypeal suture finely incised, weakly elevated and moderately angled medially. Smooth area anterior to eye three times as wide as long. Ocular canthus moderately long, finely and sparsely punctate, with a few setae. Frons with coarse and moderately dense punctures, with dense setae being bent posteriorly. Eyes large, ratio diameter/interocular width: 0.76. Antenna with ten antennomeres, club with four antennomeres and straight, as long as remaining antennomeres combined. Mentum elevated and slightly flattened anteriorly. Labrum short and almost straight anteriorly, with a transverse rim of very dense, short and robust setae.

Pronotum widest at base, lateral margins evenly convex and convergent anteriorly; anterior angles distinctly produced and sharp; posterior angles blunt, rounded at tip; anterior margin with fine, complete marginal line, weakly produced medially; surface densely and finely punctate, densely setose; anterior and lateral borders with sparse but longer setae; hypomeron carinate at base. Scutellum with fine, dense punctures and a few fine setae, on basal midline punctures less dense.

Elytra oblong, widest behind middle, striae weakly impressed, finely and densely punctate, intervals nearly flat, with fine, dense punctures, densely covered with fine, moderately long setae. Epipleural edge fine, ending at moderately curved external apical angle of elytra; epipleura densely setose, apical border with a wide membranous rim of microtrichomes (visible at magnification 100×).

Ventral surface shiny, finely and densely punctate. Metasternum with short, fine setae. Metacoxa glabrous, with a few single setae laterally. Abdominal sternites finely and densely punctate and finely setose, with a transverse row of coarse punctures each bearing a robust long seta. Mesosternum between mesocoxae as wide as mesofemur. Ratio of length of metepisternum/metacoxa: 1/1.74. Pygidium moderately convex and shiny, finely and densely punctate, without smooth midline; shortly and densely setose, with sparse long and erect setae on disc and beside the apical margin.

Legs slender; femora with two longitudinal rows of setae, finely and densely punctate. Anterior margin of metafemur acute, without adjacent serrated line; posterior margin of metafemur smooth, dorsally and ventrally, in apical half moderately widened. Metatibia wide and moderately long, widest at two thirds of metatibial length; ratio of width/length: 1/2.86; dorsal margin sharply carinate, with two groups of spines; basal group at half of metatibial length, apical group at three quarters of metatibial length; basally with a few strong short single setae; lateral face densely and coarsely punctate, shortly setose; ventral edge finely serrated, with four robust equidistant setae; medial face impunctate; apex weakly truncate interiorly near tarsal articulation. Tarsomeres ventrally with sparse, short setae; not carinate laterally, impunctate dorsally; metatarsomeres with a strongly serrated ventral ridge; first metatarsomere distinctly shorter than following two tarsomeres combined and as long as dorsal tibial spur. Protibia moderately long, bidentate; anterior claws symmetrical, basal tooth of inner claw sharply truncate at apex.

Aedeagus: Fig. 1A–C.

#### Variation.

Body length: 7.5–7.7 mm, length of elytra: 5.7–5.8 mm. Female: antennal club composed of four antennomeres, as long as the remaining antennomeres combined.

### Neoserica (s.l.) ludingensis sp. n.

Taxon classificationAnimaliaColeopteraScarabaeidae

http://zoobank.org/44F58E1B-DCD6-4423-8380-F7D6A3208BCB

Figs 1E–H , 5

#### Type material examined.

Holotype: ♂ “China West Sichuan Moximian Luding Co. 13.–18.7.94 Benes [sic]” (ZFMK). Paratypes: 2 ♂♂ “Yanzigou, Xinxing, Luding, Sichuan, 7.VIII.2004, 1560m, leg. Zhang Yong” (IZAS, ZFMK), 1 ♂ “Yanzigou, Xinxing, Luding, Sichuan, 7.VIII.2004, 1560m, leg. Bai Ming, Wan Xia” (IZAS), 1 ♂ “Hailuogou, Luding, Sichuan, 11.VIII.2004, 1900m, leg. Bai Ming” (IZAS), 1 ♂ “Huangjing, Luzhou, Sichuan, 17.VII.2002, leg. Bai Ming, Wang Jianfeng” (HBUM).

#### Description.

Body length: 7.2 mm, length of elytra: 5.3 mm, width: 4.3 mm. Body oblong, reddish brown, antennal club yellowish brown, dorsal surface shiny, densely covered with fine, semi-erect setae (Fig. 1H).

Labroclypeus subtrapezoidal, widest at base; lateral margins weakly convex and moderately convergent towards moderately rounded anterior angles; anterior margin shallowly sinuate medially; margins moderately reflexed; surface moderately elevated medially, coarsely and densely punctate, densely setose. Frontoclypeal suture finely incised, weakly elevated and moderately angled medially. Smooth area anterior to eye 2.5 times as wide as long. Ocular canthus moderately long, impunctate, with one or two single setae. Frons with coarse and moderately dense punctures, with dense setae being bent posteriorly. Eyes moderately large, ratio diameter/interocular width: 0.6. Antenna with ten antennomeres, club with four antennomeres and straight, as long as remaining antennomeres combined. Mentum elevated and slightly flattened anteriorly. Labrum short and almost straight anteriorly, with a transverse rim of very dense, short and robust setae.

Pronotum widest at base, lateral margins evenly convex and convergent anteriorly; anterior angles distinctly produced and sharp; posterior angles blunt, rounded at tip; anterior margin with fine, complete marginal line, weakly produced medially; surface densely and finely punctate, densely setose; anterior and lateral borders with sparse but longer setae; hypomeron carinate at base. Scutellum with fine, dense punctures and a few fine setae, on basal midline punctures less dense.

Elytra oblong, widest behind middle, striae weakly impressed, finely and densely punctate; intervals nearly flat, odd ones slightly convex; intervals with fine, dense punctures, densely covered with fine, moderately long setae. Epipleural edge fine, ending at moderately curved external apical angle of elytra; epipleura densely setose, apical border with a wide membranous rim of microtrichomes (visible at magnification 100×).

Ventral surface shiny, finely and densely punctate. Metasternum with short, fine setae. Metacoxa glabrous, with a few single setae laterally. Abdominal sternites finely and densely punctate, finely setose, with a transverse row of coarse punctures each bearing a long seta. Mesosternum between mesocoxae as wide as mesofemur. Ratio of length of metepisternum/metacoxa: 1/1.35. Pygidium moderately convex and shiny, finely and densely punctate, without smooth midline; shortly and densely setose, with sparse long and erect setae on disc and beside the apical margin.

Legs slender; femora with two longitudinal rows of setae, finely and densely punctate. Anterior margin of metafemur acute, without adjacent serrated line; posterior margin of metafemur smooth, dorsally and ventrally, in apical half moderately widened. Metatibia wide and moderately long, widest at two thirds of metatibial length; ratio of width/length: 1/3.2; dorsal margin sharply carinate, with two groups of spines; basal group shortly behind middle of metatibial length, apical group at three quarters of metatibial length; basally with a few strong short single setae; lateral face densely and coarsely punctate, sparsely and shortly setose; ventral edge finely serrated, with four robust equidistant setae; medial face impunctate; apex weakly truncate interiorly near tarsal articulation. Tarsomeres ventrally with sparse, short setae; not carinate laterally, impunctate dorsally; metatarsomeres with a strongly serrated ventral ridge; first metatarsomere distinctly shorter than following two tarsomeres combined and slightly longer than dorsal tibial spur. Protibia moderately long, bidentate; anterior claws symmetrical, basal tooth of inner claw sharply truncate at apex. Female unknown.

Aedeagus: Fig. 1E–G.

#### Diagnosis.

*Neoserica ludingensis* sp. n. differs from *Neoserica pilosula* by the slightly smaller eyes and by the shape of the aedeagus: the median lobe between the parameres is shorter and thicker, the left paramere is in lateral view nearly straight.

#### Etymology.

The new species is named after its occurrence in Luding county area.

#### Variation.

Body length: 7.2–7.3 mm, length of elytra: 5.3–5.4 mm, width: 4.3–4.4 mm.

### Neoserica (s.l.) weibaoshanica sp. n.

Taxon classificationAnimaliaColeopteraScarabaeidae

http://zoobank.org/BFEC3D38-CAF6-41F2-92FC-DEC18AB189F2

Figs 1I–L , 5

#### Type material examined.

Holotype: ♂ “Yunnan 2000-2800m 25.11N, 100.24E Weibaoshan mts. W slope 25–28/6.92 Vit Kubáň leg./ Coll. Milan Nikodým, Praha” (ZFMK). Paratypes: 1 ♂ Yunnan 2000–2800m 25.11N 100.24E Weibaoshan mts. W slope 25–28/6.92 Vit Kubáň leg./ Coll. Milan Nikodým, Praha” (ZFMK), 2 ♂♂, 1 ♀ “Yunnan 2000-2500m 25.42N, 100.08E Cangshan mts. E slope 21.VI.92 David Král leg.” (NMPC), 3 ♂♂ “China (N-Yunnan) Dali Bai Nat. Aut. Pref., 1 km W of Dali old town, creek valley at foothill of Diancang Shan, 2170m, 25°41.9'N, 100°08.4'E (along creek under stones, plant roots, in soil) 19./23.VI.2005 D.W. Wrase [13A]” (ZFMK), 1 ♂ “Yunnan 2500–2700m 25.58N, 100.21E Jizu Shan 6–10.7. Vit Kubáň leg. 1994” (CPPB), 1 ♂ “China- Yunnan prov. 22-27 July 1998 Dali old tower env. Zd. Jindra lgt.” (ZFMK), 2 ♂♂, 3 ♀♀ “China, N.Yunnan, env. Xiaguan, 2400m, 29.vii.2002, leg. S. Murzin, I. Shokhin” (CPPB, ZFMK).

#### Description.

Body length: 7.5 mm, length of elytra: 5.0 mm, width: 3.8 mm. Body oblong, reddish brown, antennal club yellowish brown, dorsal surface shiny, densely covered with fine, semi-erect setae (Fig. 1L).

Labroclypeus short and subtrapezoidal, widest at base; lateral margins weakly convex and moderately convergent towards strongly rounded anterior angles; anterior margin shallowly sinuate medially; margins moderately reflexed; surface moderately elevated medially, coarsely and finely but densely punctate, sparsely setose. Frontoclypeal suture finely incised, weakly elevated and moderately angled medially. Smooth area anterior to eye 2.5 times as wide as long. Ocular canthus narrow and moderately long, sparsely punctate, with one or two single setae. Frons with coarse and moderately dense punctures, with dense setae being bent posteriorly. Eyes large, ratio diameter/interocular width: 0.76. Antenna with ten antennomeres, club with four antennomeres and straight, 1.2 times as long as remaining antennomeres combined. Mentum elevated and slightly flattened anteriorly. Labrum short and almost straight anteriorly, with a transverse rim of very dense, short and robust setae.

Pronotum widest at base, lateral margins evenly convex and moderately convergent anteriorly; anterior angles moderately produced and sharp; posterior angles blunt, rounded at tip; anterior margin with robust, complete marginal line, weakly produced medially; surface densely and finely punctate, densely setose; anterior and lateral borders with sparse but longer setae; hypomeron carinate at base. Scutellum with fine, dense punctures and a few fine setae.

Elytra oblong, widest behind middle, striae weakly impressed, finely and densely punctate; intervals nearly flat, odd ones slightly convex; intervals with fine, dense punctures, densely covered with fine, moderately long setae. Epipleural edge fine, ending at moderately curved external apical angle of elytra; epipleura densely setose, apical border with a wide membranous rim of microtrichomes (visible at magnification 100×).

Ventral surface shiny, finely and densely punctate. Metasternum with short, fine setae. Metacoxa glabrous, with a few single setae laterally. Abdominal sternites finely and densely punctate, finely setose, with a transverse row of coarse punctures each bearing a long seta. Mesosternum between mesocoxae as wide as mesofemur. Ratio of length of metepisternum/metacoxa: 1/1.59. Pygidium moderately convex and shiny, finely and densely punctate, without smooth midline; with dense, moderately long setae on disc and beside the apical margin.

Legs slender; femora with two longitudinal rows of setae, finely and densely punctate. Anterior margin of metafemur acute, without adjacent serrated line; posterior margin of metafemur smooth, dorsally and ventrally, in apical half moderately widened, dorsal posterior margin with dense and thick, evenly long setae. Metatibia wide and moderately long, widest at two thirds of metatibial length; ratio of width/length: 1/3.0; dorsal margin sharply carinate, with two groups of spines; basal group shortly behind middle of metatibial length, apical group at three quarters of metatibial length; in basal half with a continuously serrated line and some single punctures each bearing a short seta; lateral face densely and coarsely punctate, densely and shortly setose; ventral edge finely serrated, with four robust equidistant setae; medial face impunctate; apex weakly truncate interiorly near tarsal articulation. Tarsomeres ventrally with sparse, short setae; not carinate laterally, impunctate dorsally; metatarsomeres with a strongly serrated ventral ridge; first metatarsomere distinctly shorter than following two tarsomeres combined and slightly longer than dorsal tibial spur. Protibia moderately long, bidentate; anterior claws symmetrical, basal tooth of inner claw sharply truncate at apex.

Aedeagus: Fig. 1I–K.

#### Diagnosis.

*Neoserica weibaoshanica* sp. n. differs from *Neoserica pilosula* by the longer antennal club, the shorter labroclypeus, and the shape of trifid median lobe of aedeagus bearing a long filiform process on the left side shortly after the base and a short spine on the right side before the apex.

#### Etymology.

The new species is named after its type locality in Weibaoshan Mts.

#### Variation.

Body length: 6.9–7.9 mm, length of elytra: 4.4–5.5 mm, width: 3.6–4.4 mm. Female: antennal club composed of four antennomeres, as long as the remaining antennomeres combined.

### Neoserica (s.l.) tianeana sp. n.

Taxon classificationAnimaliaColeopteraScarabaeidae

http://zoobank.org/450EBD39-435E-4517-A66B-F5352E46E9BF

Figs 2A–D , 5

#### Type material examined.

Holotype: ♂ “Dashan Forestry Farm, Tian’e, Guangxi, 3.VIII.2002, 1100m, leg. Jiang Guofang” (IZAS).

#### Description.

**Figure 2.**
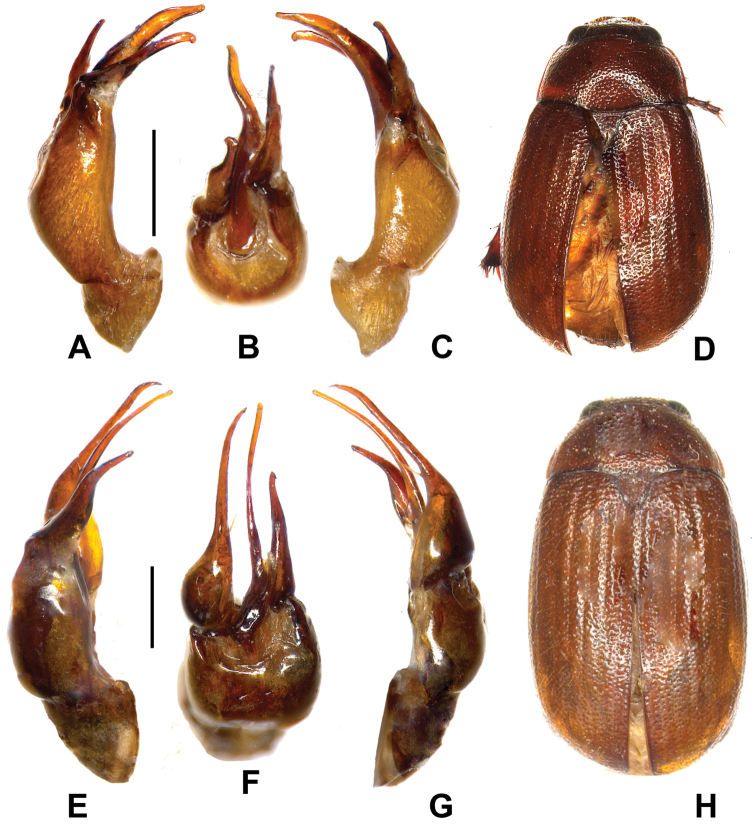
**A–D** *Neoserica tianeana* sp. n. (holotype) **E–H** *Neoserica shennongjiaensis* sp. n. (holotype). **A, E** aedeagus, left side lateral view **C, G** aedeagus, right side lateral view **B, F** parameres, dorsal view **D, H** habitus. Scale: 0.5 mm, habitus not to scale.

Body length: 7.2 mm, length of elytra: 5.2 mm, width: 3.9 mm. Body oblong, reddish brown, antennal club yellowish brown, dorsal surface shiny, densely covered with fine, semi-erect setae (pilosity partly abraded; Fig. 2D).

Labroclypeus subtrapezoidal, widest at base; lateral margins weakly convex and moderately convergent towards moderately rounded anterior angles; anterior margin shallowly sinuate medially; margins moderately reflexed; surface moderately elevated medially, coarsely and finely, densely punctate, densely setose. Frontoclypeal suture finely incised, weakly elevated and moderately angled medially. Smooth area anterior to eye three times as wide as long. Ocular canthus moderately long, impunctate, with two or three setae. Frons with coarse and dense punctures, with dense setae being bent posteriorly. Eyes large, ratio diameter/interocular width: 0.75. Antenna with ten antennomeres, club with four antennomeres and straight, as long as remaining antennomeres combined. Mentum elevated and slightly flattened anteriorly. Labrum short and almost straight anteriorly, with a transverse rim of very dense, short and robust setae.

Pronotum widest at base, lateral margins weakly convex and convergent anteriorly; anterior angles distinctly produced and moderately sharp; posterior angles blunt, rounded at tip; anterior margin with fine, complete marginal line, weakly produced medially; surface densely and finely punctate, densely setose; anterior and lateral borders with sparse but longer setae; hypomeron carinate at base. Scutellum with fine, dense punctures and a few fine setae.

Elytra oblong, widest behind middle, striae weakly impressed, finely and densely punctate; intervals nearly flat, odd ones slightly convex; intervals with fine, dense punctures, densely covered with fine, moderately long setae. Epipleural edge fine, ending at moderately curved external apical angle of elytra; epipleura densely setose, apical border with a wide membranous rim of microtrichomes (visible at magnification 100×).

Ventral surface shiny, finely and densely punctate. Metasternum with short, fine setae. Metacoxa glabrous, with a few single setae laterally. Abdominal sternites finely and densely punctate, finely setose, with a transverse row of coarse punctures each bearing a long seta. Mesosternum between mesocoxae as wide as mesofemur. Ratio of length of metepisternum/metacoxa: 1/1.73. Pygidium strongly convex and shiny, finely and densely punctate, without smooth midline; shortly and densely setose, with sparse long and erect setae on disc and beside the apical margin.

Legs slender; femora with two longitudinal rows of setae, finely and densely punctate. Anterior margin of metafemur acute, without adjacent serrated line; posterior margin of metafemur smooth, dorsally and ventrally, in apical half moderately widened. Metatibia wide and moderately long, widest at middle; ratio of width/length: 1/2.7; dorsal margin sharply carinate, with two groups of spines; basal group at middle, apical group at three quarters of metatibial length; in basal half with a few strong and short single setae in coarse punctures beside a undulated serrated line; lateral face densely and coarsely punctate, densely and shortly setose; ventral edge finely serrated, with four robust equidistant setae; medial face impunctate; apex weakly truncate interiorly near tarsal articulation. Meso- and metatarsomeres of holotype also missing. Protibia moderately long, bidentate; anterior claws symmetrical, basal tooth of inner claw sharply truncate at apex. Female unknown.

Aedeagus: Fig. 2A–C.

#### Diagnosis.

*Neoserica tianeana* sp. n. differs from *Neoserica pilosula* and the other previous species by the long, non-spherical right paramere being subequal in length to the left one.

#### Etymology.

The new species is named after the type locality, Tian’e.

### Neoserica (s.l.) shennongjiaensis sp. n.

Taxon classificationAnimaliaColeopteraScarabaeidae

http://zoobank.org/CF4AEAAC-BB7A-4CE7-8D8E-4C36CB51E38C

Figs 2E–H , 5

#### Type material examined.

Holotype: ♂ “Honghua, Shennongjia, Hubei, 26.VII.1980, 900, leg. Yu Peiyu” (IZAS). Paratypes: 1 ♀ “Honghua, Shennongjia, Hubei, 26.VII.1980, 900, leg. Yu Peiyu/ LW-617” (IZAS), 1 ♂ “Mts. Zhongtiaoshan, Shanxi, 30.VII.1995, 550m, leg. Li Wenzhu” (IZAS), 1 ♂ “Hetouzhai, Jinping, Yunnan, 15.V.1956, 1700m, leg. Huang Keren” (ZFMK), 1 ♂ “Dashahe, Daozhen, Guizhou, 17–21.VIII.2004, leg. Yang Xiujuan, Hua Huiran” (HBUM), 1 ♂ “Mt. Baiyunshan, Songxian County, Henan, 14–17.VIII.2008, leg. Ren Guodong, Wu Qiqi etc.” (HBUM).

#### Description.

Body length: 7.5 mm, length of elytra: 5.7 mm, width: 4.2 mm. Body oblong, reddish brown, antennal club yellowish brown, dorsal surface shiny, densely covered with fine, semi-erect setae (Fig. 2H).

Labroclypeus subtrapezoidal, widest at base; lateral margins weakly convex and moderately convergent towards strongly rounded anterior angles; anterior margin distinctly sinuate medially; margins moderately reflexed; surface moderately elevated medially, coarsely and finely but densely punctate, sparsely setose. Frontoclypeal suture finely incised, weakly elevated and moderately angled medially. Smooth area anterior to eye 2.5 times as wide as long. Ocular canthus narrow and moderately long, sparsely punctate, with one or two single setae. Frons with coarse and moderately dense punctures, with dense setae being bent posteriorly. Eyes large, ratio diameter/interocular width: 0.73. Antenna with ten antennomeres, club with four antennomeres and straight, 1.2 times as long as remaining antennomeres combined. Mentum elevated and slightly flattened anteriorly. Labrum short and almost straight anteriorly, with a transverse rim of very dense, short and robust setae.

Pronotum widest at base, lateral margins evenly convex and moderately convergent anteriorly; anterior angles moderately produced and sharp; posterior angles blunt, rounded at tip; anterior margin with fine, complete marginal line, weakly produced medially; surface densely and finely punctate, densely setose; anterior and lateral borders with sparse but longer setae; hypomeron carinate at base. Scutellum with fine, dense punctures and a few fine setae.

Elytra oblong, widest behind middle, striae weakly impressed, finely and densely punctate; intervals nearly flat, odd ones slightly convex; intervals with fine, dense punctures, densely covered with fine, moderately long setae. Epipleural edge fine, ending at moderately curved external apical angle of elytra; epipleura densely setose, apical border with a wide membranous rim of microtrichomes (visible at magnification 100×).

Ventral surface shiny, finely and densely punctate. Metasternum with short, fine setae. Metacoxa glabrous, with a few single setae laterally. Abdominal sternites finely and densely punctate, finely setose, with a transverse row of coarse punctures each bearing a long seta. Mesosternum between mesocoxae as wide as mesofemur. Ratio of length of metepisternum/metacoxa: 1/1.62. Pygidium weakly convex and shiny, finely and densely punctate, without smooth midline; with dense, long setae on disc and beside the apical margin.

Legs slender; femora with two longitudinal rows of setae, finely and densely punctate. Anterior margin of metafemur acute, without adjacent serrated line; posterior margin of metafemur smooth, dorsally and ventrally, in apical half moderately widened, dorsal posterior margin with fine setae. Metatibia wide and moderately long, widest at two thirds of metatibial length; ratio of width/length: 1/2.7; dorsal margin sharply carinate, with two groups of spines; basal group shortly behind middle of metatibial length, apical group at three quarters of metatibial length; in basal half with a continuously serrated line and some single punctures each bearing a short seta; lateral face densely and coarsely punctate, densely and shortly setose; ventral edge finely serrated, with four robust equidistant setae; medial face impunctate; apex weakly truncate interiorly near tarsal articulation. Tarsomeres ventrally with sparse, short setae; not carinate laterally, impunctate dorsally; metatarsomeres with a strongly serrated ventral ridge; metatarsomeres 2-5 and dorsal tibial spur of holotype also missing. Protibia moderately long, bidentate; anterior claws symmetrical, basal tooth of inner claw sharply truncate at apex.

Aedeagus: Fig. 2E–G.

#### Diagnosis.

*Neoserica shennongjiaensis* sp. n. differs from *Neoserica tianeana* sp. n. by the significantly longer right paramere.

#### Etymology.

The new species is named after the type locality, Shennongjia.

#### Variation.

Body length: 7.5–8.0 mm, length of elytra: 5.1–5.7 mm, width: 3.8–4.2 mm. First metatarsomere distinctly shorter than following two tarsomeres combined and slightly longer than dorsal tibial spur. Female: antennal club composed of four antennomeres, as long as the remaining antennomeres combined.

### Neoserica (s.l.) lincangensis sp. n.

Taxon classificationAnimaliaColeopteraScarabaeidae

http://zoobank.org/9B76942F-5E0D-4141-A78A-82295FC18F49

Figs 3A–D , 5

#### Type material examined.

Holotype: ♂ “Yunnan, Lincang, Mt. Wulaoshan, 2010-VII-31, 23.90648N, 100.15944E, 1807m/ LW-1324” (IZAS). Paratype: 1 ♀ “Yunnan, Lincang, Mt. Wulaoshan, 2010-VII-31, 23.90648N, 100.15944E, 1807m/ LW-1324b” (ZFMK).

#### Description.

**Figure 3.**
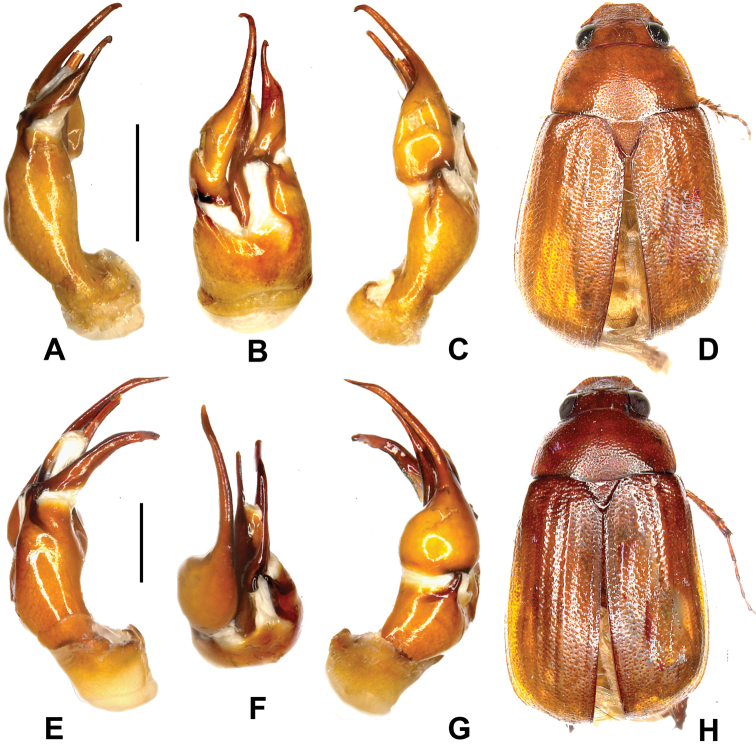
**A–D** *Neoserica lincangensis* sp. n. (holotype) **E–H** *Neoserica rangshuiensis* sp. n. (holotype). **A, E** edeagus, left side lateral view **C, G** aedeagus, right side lateral view **B, F** parameres, dorsal view **D, H** habitus. Scale: 0.5 mm, habitus not to scale.

Body length: 6.7 mm, length of elytra: 4.8 mm, width: 3.8 mm. Body oblong, reddish brown, antennal club yellowish brown, dorsal surface shiny, densely covered with fine, semi-erect setae (Fig. 3D).

Labroclypeus subtrapezoidal, widest at base; lateral margins weakly convex and moderately convergent towards strongly rounded anterior angles; anterior margin distinctly sinuate medially; margins moderately reflexed; surface moderately elevated medially, coarsely and finely but densely punctate, sparsely setose. Frontoclypeal suture finely incised, weakly elevated and moderately angled medially. Smooth area anterior to eye twice as wide as long. Ocular canthus narrow and moderately long, sparsely punctate, with two long setae. Frons with coarse and moderately dense punctures, with dense setae being bent posteriorly. Eyes large, ratio diameter/interocular width: 0.74. Antenna with ten antennomeres, club with four antennomeres and straight, as long as remaining antennomeres combined. Mentum elevated and slightly flattened anteriorly. Labrum short and almost straight anteriorly, with a transverse rim of very dense, short and robust setae.

Pronotum widest at base, lateral margins evenly convex and moderately convergent anteriorly; anterior angles moderately produced and sharp; posterior angles blunt, rounded at tip; anterior margin with fine, complete marginal line, weakly produced medially; surface densely and finely punctate, around midline punctures very dense, partly fusing with each other transversely, densely setose; anterior and lateral borders with sparse but longer setae; hypomeron carinate at base. Scutellum with fine, dense punctures and a few fine setae.

Elytra oblong, widest behind middle, striae weakly impressed, finely and densely punctate; intervals nearly flat, odd ones slightly convex; intervals with fine, dense punctures, densely covered with fine, moderately long setae. Epipleural edge fine, ending at moderately curved external apical angle of elytra; epipleura densely setose, apical border with a wide membranous rim of microtrichomes (visible at magnification 100×).

Ventral surface shiny, finely and densely punctate. Metasternum with short, fine setae. Metacoxa glabrous, with a few single setae laterally. Abdominal sternites finely and densely punctate, finely setose, with a transverse row of coarse punctures each bearing a long seta. Mesosternum between mesocoxae as wide as mesofemur. Ratio of length of metepisternum/metacoxa: 1/1.57. Pygidium weakly convex and shiny, finely and densely punctate, without smooth midline; with dense, long setae on disc and beside the apical margin.

Legs slender; femora with two longitudinal rows of setae, finely and densely punctate. Anterior margin of metafemur acute, without adjacent serrated line; posterior margin of metafemur smooth, dorsally and ventrally, in apical half moderately widened, dorsal posterior margin with fine setae. Metatibia wide and moderately long, widest at two thirds of metatibial length; ratio of width/length: 1/2.7; dorsal margin sharply carinate, with two groups of spines; basal group shortly behind middle of metatibial length, apical group at three quarters of metatibial length; in basal half with a undulated, nearly continuously serrated line and beside it some single punctures each bearing a short seta; lateral face moderately densely and coarsely punctate, shortly setose; ventral edge finely serrated, with four robust equidistant setae; medial face impunctate; apex weakly truncate interiorly near tarsal articulation. Tarsomeres ventrally with sparse, short setae; not carinate laterally, impunctate dorsally; metatarsomeres with a strongly serrated ventral ridge; first metatarsomere distinctly shorter than following two tarsomeres combined and slightly longer than dorsal tibial spur. Protibia moderately long, bidentate; anterior claws symmetrical, basal tooth of inner claw sharply truncate at apex.

Aedeagus: Fig. 3A–C.

#### Diagnosis.

*Neoserica lincangensis* sp. n. is most similar to *Neoserica shennongjiaensis* sp. n. but differs from it by the shorter antennal club and the shape of the parameres: the right paramere is basally strongly enlarged and abruptly curved at apex.

#### Etymology.

The new species is named after its occurrence in the Lincang county.

#### Variation.

Body length: 6.4–6.7 mm, length of elytra: 4.5–4.8 mm, width: 3.0–3.8 mm. Female: antennal club composed of four antennomeres, as long as the remaining antennomeres combined.

### Neoserica (s.l.) rangshuiensis sp. n.

Taxon classificationAnimaliaColeopteraScarabaeidae

http://zoobank.org/3104170D-10E8-4C3C-898F-CEF1C2CB7D42

Figs 3E–H , 5

#### Type material examined.

Holotype: ♂ “Guizhou, Kuankuoshui Nature Reserve, Rangshui, 2010-VIII-15, 1527m, 28.22N, 107.19E daytime/ LW-1380” (IZAS). Paratype: 1 ♂ “Guizhou, Zunyi, Kuankuoshui Nature Reserve, Rangshui, 2010-VIII-16, 860m/ LW-1032” (ZFMK).

#### Description.

Body length: 7.3 mm, length of elytra: 5.2 mm, width: 3.9 mm. Body oblong, reddish brown, antennal club yellowish brown, dorsal surface shiny, densely covered with fine and short, semi-erect setae (in part abraded; Fig. 3H).

Labroclypeus subtrapezoidal, widest at base; lateral margins weakly convex and moderately convergent towards moderately rounded anterior angles; anterior margin shallowly sinuate medially; margins moderately reflexed; surface convexly elevated medially, coarsely and finely but densely punctate, sparsely setose. Frontoclypeal suture finely incised, weakly elevated and moderately angled medially. Smooth area anterior to eye 2.5 times as wide as long. Ocular canthus narrow and moderately long, sparsely punctate, with a single short terminal seta. Frons with coarse and sparse punctures, with numerous setae being bent posteriorly. Eyes large, ratio diameter/interocular width: 0.78. Antenna with ten antennomeres, club with four antennomeres and straight, 1.2 times as long as remaining antennomeres combined. Mentum elevated and slightly flattened anteriorly. Labrum short and almost straight anteriorly, with a transverse rim of very dense, short and robust setae.

Pronotum widest at base, lateral margins nearly straight and convergent, slightly convex anteriorly and moderately convergent towards moderately produced and sharp anterior angles; posterior angles blunt, rounded at tip; anterior margin with fine, complete marginal line, weakly produced medially; surface densely and finely punctate, except on disc (probably abraded) densely setose; anterior and lateral borders with sparse but longer setae; hypomeron carinate at base. Scutellum with fine, dense punctures and a few fine setae.

Elytra oblong, widest behind middle, striae weakly impressed, finely and densely punctate; intervals nearly flat, odd ones slightly convex; intervals with fine, dense punctures, punctures on odd intervals concentrated along striae, densely covered with fine, moderately long setae. Epipleural edge fine, ending at moderately curved external apical angle of elytra; epipleura densely setose, apical border with a wide membranous rim of microtrichomes (visible at magnification 100×).

Ventral surface shiny, finely and densely punctate. Metasternum with short, fine setae. Metacoxa glabrous, with a few single setae laterally. Abdominal sternites finely and densely punctate, finely setose, with a transverse row of coarse punctures each bearing a long seta. Mesosternum between mesocoxae as wide as mesofemur. Ratio of length of metepisternum/metacoxa: 1/1.57. Pygidium strongly convex and shiny, finely and densely punctate, without smooth midline; with dense, long setae on disc and beside the apical margin.

Legs slender; femora with two longitudinal rows of setae, finely and densely punctate. Anterior margin of metafemur acute, without adjacent serrated line; posterior margin of metafemur smooth, dorsally and ventrally, in apical half moderately widened, dorsal posterior margin with fine setae. Metatibia wide and moderately long, widest at two thirds of metatibial length; ratio of width/length: 1/2.7; dorsal margin sharply carinate, with two groups of spines; basal group shortly behind middle of metatibial length, apical group at three quarters of metatibial length; in basal half with a undulated, nearly continuously serrated line and beside it single coarse punctures each bearing a short robust seta; lateral face moderately densely and coarsely punctate, shortly setose; ventral edge finely serrated, with four robust equidistant setae; medial face impunctate; apex weakly truncate interiorly near tarsal articulation. Tarsomeres ventrally with sparse, short setae; not carinate laterally, impunctate dorsally; metatarsomeres missing in holo- and paratype. Protibia moderately long, bidentate; anterior claws symmetrical, basal tooth of inner claw sharply truncate at apex.

Aedeagus: Fig. 3E–G.

#### Diagnosis.

*Neoserica rangshuiensis* sp. n. is most similar to *Neoserica lincangensis* sp. n. but differs from it by the slightly longer antennal club and the shape of the parameres: the right paramere is only in the basal third strongly enlarged (not in basal half as in *Neoserica lincangensis* sp. n.) and slightly bent at the apex only (not curved); the left paramere is evenly curved (not straight or double-bent).

#### Etymology.

The new species is named after its type locality, Rangshui.

#### Variation.

Body length: 7.3–7.4 mm, length of elytra: 5.2–5.3 mm. Metatarsomeres of the paratype with a strongly serrated ventral ridge; first metatarsomere distinctly shorter than the following two tarsomeres combined and slightly longer than the dorsal tibial spur.

### Neoserica (s.l.) lushuiana sp. n.

Taxon classificationAnimaliaColeopteraScarabaeidae

http://zoobank.org/B8FBBBE0-C613-4BA3-B62C-B3C847FFD79C

Figs 4A–D , 5

#### Type material examined.

Holotype: ♂ “Lushui, Yunnan, 9.VI.1981, 1810m, leg. Wang Shuyong, No.17” (IZAS).

#### Description.

**Figure 4.**
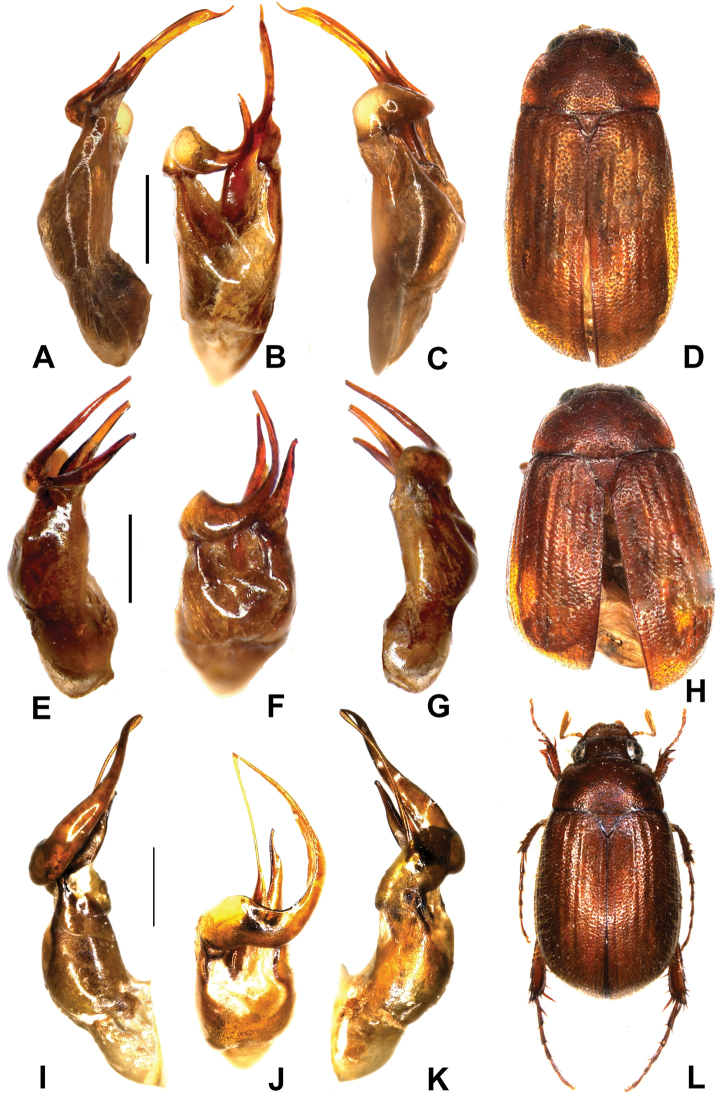
**A–D** *Neoserica lushuiana* sp. n. (holotype) **E–H** *Neoserica emeishanensis* sp. n. (holotype) **I–L** *Neoserica curvipenis* sp. n. (holotype). **A, E, I** aedeagus, left side lateral view **C, G, K** aedeagus, right side lateral view **B, F, J** parameres, dorsal view **D, H, L** habitus. Scale: 0.5 mm, habitus not to scale.

Body length: 6.2 mm, length of elytra: 4.5 mm, width: 3.2 mm. Body oblong, reddish brown, antennal club yellowish brown, dorsal surface shiny, densely covered with fine, semi-erect setae (Fig. 4D).

Labroclypeus subtrapezoidal, widest at base; lateral margins weakly convex and moderately convergent towards moderately rounded anterior angles; anterior margin shallowly sinuate medially; margins moderately reflexed; surface moderately convex medially, coarsely and finely but densely punctate, sparsely setose. Frontoclypeal suture finely incised, weakly elevated and moderately angled medially. Smooth area anterior to eye 2.5 times as wide as long. Ocular canthus narrow and moderately long, sparsely punctate, with a single short terminal seta. Frons with coarse and dense punctures, with dense setae being bent posteriorly. Eyes large, ratio diameter/interocular width: 0.71. Antenna with ten antennomeres, club with four antennomeres and straight, 1.2 times as long as remaining antennomeres combined. Mentum elevated and slightly flattened anteriorly. Labrum short and almost straight anteriorly, with a transverse rim of very dense, short and robust setae.

Pronotum widest at posterior third, lateral margins evenly convex, moderately convergent posteriorly and towards moderately produced and sharp anterior angles; posterior angles blunt, rounded at tip; anterior margin with fine, complete marginal line, weakly produced medially; surface densely and finely punctate, densely setose; anterior and lateral borders with dense, long setae; hypomeron carinate at base. Scutellum small, with fine, dense punctures and a few fine setae.

Elytra oblong, widest behind middle, striae weakly impressed, finely and densely punctate; intervals flat, odd ones slightly convex; intervals with fine, dense punctures, punctures on odd intervals concentrated along striae, densely covered with fine, moderately long setae. Epipleural edge fine, ending at moderately curved external apical angle of elytra; epipleura densely setose, apical border with a wide membranous rim of microtrichomes (visible at magnification 100×).

Ventral surface shiny, finely and densely punctate. Metasternum with short, fine setae. Metacoxa glabrous, with a few single setae laterally. Abdominal sternites finely and densely punctate, finely setose, with a transverse row of coarse punctures each bearing a long seta. Mesosternum between mesocoxae as wide as mesofemur. Ratio of length of metepisternum/metacoxa: 1/1.55. Pygidium moderately convex and shiny, finely and densely punctate, without smooth midline; with dense, long setae on disc and beside the apical margin.

Legs slender; femora with two longitudinal rows of setae, finely and densely punctate. Anterior margin of metafemur acute, without adjacent serrated line; posterior margin of metafemur smooth, dorsally and ventrally, in apical half moderately widened, dorsal posterior margin with sparse, fine setae. Metatibia wide and moderately long, widest at two thirds of metatibial length; ratio of width/length: 1/3.0; dorsal margin sharply carinate, with two groups of spines; basal group shortly behind middle of metatibial length, apical group at three quarters of metatibial length; in basal half with a slightly undulated, nearly continuously serrated line and beside it single coarse punctures each bearing a short robust seta; lateral face moderately densely and coarsely punctate, shortly setose; ventral edge finely serrated, with four robust equidistant setae; medial face impunctate; apex weakly truncate interiorly near tarsal articulation. Tarsomeres ventrally with sparse, short setae; not carinate laterally, impunctate dorsally; metatarsomeres with a strongly serrated ventral ridge; first metatarsomere distinctly shorter than following two tarsomeres combined and slightly longer than dorsal tibial spur. Protibia moderately long, bidentate; anterior claws symmetrical, basal tooth of inner claw sharply truncate at apex. Female unknown.

Aedeagus: Fig. 4A–C.

#### Diagnosis.

*Neoserica lushuiana* sp. n. differs from all other species of the *Neoserica pilosula* group by having the right paramere strongly curved externally (in dorsal view).

#### Etymology.

The new species is named after its type locality, Lushui.

### Neoserica (s.l.) emeishanensis sp. n.

Taxon classificationAnimaliaColeopteraScarabaeidae

http://zoobank.org/FD694F41-71E6-4D4D-922F-1B7C45E4D001

Figs 4E–H , 5

#### Type material examined.

Holotype: ♂ “Qingyin’ge, Mts. Emeishan, Sichuan, 21.IX.1957, 800–1000m, leg. Zhu Fuxing/ LW-670” (IZAS). Paratypes: 2 ♂♂ “Qingyin’ge, Mts. Emeishan, Sichuan, 21.IX.1957, 800-1000m, leg. Zhu Fuxing” (IZAS, ZFMK).

#### Description.

Body length: 6.6 mm, length of elytra: 5.1 mm, width: 4.0 mm. Body oblong, reddish brown, antennal club yellowish brown, dorsal surface shiny, elytra densely covered with fine, semi-erect setae; setae on head and pronotum abraded in type specimens (Fig. 4H).

Labroclypeus subtrapezoidal, widest at base; lateral margins weakly convex and moderately convergent towards moderately rounded anterior angles; anterior margin distinctly sinuate medially; margins moderately reflexed; surface moderately convex medially, coarsely and finely but densely punctate, sparsely setose. Frontoclypeal suture finely incised, weakly elevated and moderately angled medially. Smooth area anterior to eye twice as wide as long. Ocular canthus narrow and moderately long, sparsely punctate, glabrous. Frons with coarse and dense punctures, with a few moderately long setae beside eyes. Eyes moderately large, ratio diameter/interocular width: 0.68. Antenna with ten antennomeres, club with four antennomeres and straight, 1.2 times as long as remaining antennomeres combined. Mentum elevated and slightly flattened anteriorly. Labrum short and almost straight anteriorly, with a transverse rim of very dense, short and robust setae.

Pronotum widest at base, lateral margins evenly convex, moderately convergent towards moderately produced and sharp anterior angles; posterior angles blunt, rounded at tip; anterior margin with fine, complete marginal line, weakly produced medially; surface densely and finely punctate, sparsely setose; anterior and lateral borders with sparse, long setae; hypomeron carinate at base. Scutellum small, with fine, dense punctures and a few fine setae.

Elytra oblong, widest behind middle, striae weakly impressed, finely and densely punctate; intervals flat, odd ones slightly convex; intervals with fine, dense punctures, punctures on odd intervals concentrated along striae, densely covered with fine, moderately long setae. Epipleural edge fine, ending at moderately curved external apical angle of elytra; epipleura densely setose, apical border with a wide membranous rim of microtrichomes (visible at magnification 100×).

Ventral surface shiny, finely and densely punctate. Metasternum with short, fine setae. Metacoxa glabrous, with a few single setae laterally. Abdominal sternites finely and densely punctate, finely setose, with a transverse row of coarse punctures each bearing a long seta. Mesosternum between mesocoxae as wide as mesofemur. Ratio of length of metepisternum/metacoxa: 1/1.61. Pygidium moderately convex and shiny, shortly and densely punctate, without smooth midline; with dense, long setae beside the apical margin.

Legs slender; femora with two longitudinal rows of setae, finely and densely punctate. Anterior margin of metafemur acute, without adjacent serrated line; posterior margin of metafemur smooth, dorsally and ventrally, in apical half moderately widened, dorsal posterior margin with sparse, fine setae. Metatibia wide and moderately long, widest at two thirds of metatibial length; ratio of width/length: 1/2.85; dorsal margin sharply carinate, with two groups of spines; basal group shortly behind middle of metatibial length, apical group at three quarters of metatibial length; in basal half with a slightly undulated, nearly continuously serrated line and beside it single coarse punctures each bearing a short robust seta; lateral face moderately densely and coarsely punctate, shortly setose; ventral edge finely serrated, with four robust equidistant setae; medial face impunctate; apex weakly truncate interiorly near tarsal articulation. Tarsomeres ventrally with sparse, short setae; not carinate laterally, impunctate dorsally; metatarsomeres missing in holotype. Protibia moderately long, bidentate; anterior claws symmetrical, basal tooth of inner claw sharply truncate at apex. Female unknown.

Aedeagus: Fig. 4E–G.

#### Diagnosis.

*Neoserica emeishanensis* sp. n. is most similar to *Neoserica lushuiana* sp. n. but differs from it by the longer right paramere (in dorsal view) being much longer than the phallobase width.

#### Etymology.

The new species is named after its type locality, Mt. Emeishan.

#### Variation.

Body length: 6.6–7.0 mm, length of elytra: 5.1–5.4 mm, width: 4.0–4.2 mm.

#### Remarks.

Metatarsomeres are missing in all paratypes.

### Neoserica (s.l.) curvipenis sp. n.

Taxon classificationAnimaliaColeopteraScarabaeidae

http://zoobank.org/8AB9299A-AACB-4B78-9364-E287FF85F2B4

Figs 4I–L , 5

#### Type material examined.

Holotype: ♂ “Yunnan 2000–3000m 27.20N, 100.11E Habashan mts. SE slope 10-13/7. Vit Kubáň leg. 92/ [ex] coll. Milan Nikodým, Praha” (ZFMK). Paratypes: 1 ♀ “Yunnan 2000-3000m 27.20N, 100.11E Habashan mts. SE slope 10-13/7. Vit Kubáň leg. 92/ [ex] coll. Milan Nikodým, Praha” (ZFMK), 1 ♂ “Yunnan cca 2000m 27.15N, 100.09E Hutiao gorge Jinsha r. 18-22/7. leg. Vit Kubáň leg. 92” (ZFMK), 1 ♂ “China West Sichuan Moximian Luding Co. 13.–18.7.94 Benes” (ZFMK), 1 ♂ “China; Yunnan prov.; Daju – 50km N Lijiang; 27.21N, 100.19E; S. Bečvář leg.; 21.–27.vi.1993” (CPPB), 2 ♂♂, 1 ♀ “China Yunnan 2000-3000m 27°20’N, 100°11’E Habashan Mts. SE slope, 10.[-]13.7.1992. D. Král lgt.” (NMPC), 1 ♂ “Yunnan ca. 2000m 27.15N, 100.09E Hutiao gorge Jinsha r. 18–22.7.92 leg. David Král” (NMPC), 1 ♂ “Qingyinge, Emeishan, Sichuan, 22.IX.1957, 800–1000m, leg. Zhu Fuxing” (IZAS).

#### Description.

Body length: 7.6 mm, length of elytra: 5.2 mm, width: 4.0 mm. Body oblong, reddish brown, antennal club yellowish brown, dorsal surface shiny, densely covered with fine, semi-erect setae (Fig. 4L).

Labroclypeus subtrapezoidal, widest at base; lateral margins convex and convergent towards moderately rounded anterior angles; anterior margin distinctly sinuate medially; margins moderately reflexed; surface weakly convex medially, coarsely and finely but densely punctate, densely setose. Frontoclypeal suture finely incised, weakly elevated and moderately angled medially. Smooth area anterior to eye 1.5 times as wide as long. Ocular canthus narrow and moderately long, sparsely punctate, with a few long seta. Frons with coarse and dense punctures mixed with sparse, fine ones, with dense setae being bent posteriorly. Eyes large, ratio diameter/interocular width: 0.71. Antenna with ten antennomeres, club with four antennomeres and straight, as long as remaining antennomeres combined. Mentum elevated and slightly flattened anteriorly. Labrum short and almost straight anteriorly, with a transverse rim of very dense, short and robust setae.

Pronotum widest at base, lateral margins evenly convex and moderately convergent towards moderately produced and sharp anterior angles; posterior angles blunt, rounded at tip; anterior margin with fine, complete marginal line, weakly produced medially; surface densely and finely punctate, densely setose; anterior and lateral borders with dense, long setae; hypomeron carinate at base. Scutellum small, with fine, dense punctures and dense, fine setae.

Elytra oblong, widest behind middle, striae weakly impressed, finely and densely punctate; intervals flat, odd ones slightly convex; intervals with fine, dense punctures, punctures on odd intervals concentrated along striae, densely covered with fine, moderately long setae. Epipleural edge fine, ending at moderately curved external apical angle of elytra; epipleura densely setose, apical border with a wide membranous rim of microtrichomes (visible at magnification 100×).

Ventral surface shiny, finely and densely punctate. Metasternum with short, fine setae. Metacoxa glabrous, with a few single setae laterally. Abdominal sternites finely and densely punctate, finely setose, with a transverse row of coarse punctures each bearing a long seta. Mesosternum between mesocoxae as wide as mesofemur. Ratio of length of metepisternum/metacoxa: 1/1.74. Pygidium moderately convex and shiny, finely and densely punctate, without smooth midline; with dense, long setae on disc and beside the apical margin.

Legs slender; femora with two longitudinal rows of setae, finely and densely punctate. Anterior margin of metafemur acute, without adjacent serrated line; posterior margin of metafemur smooth, dorsally and ventrally, in apical half moderately widened, dorsal posterior margin with sparse, fine setae. Metatibia wide and moderately long, widest at two thirds of metatibial length; ratio of width/length: 1/3.33; dorsal margin sharply carinate, with two groups of spines; basal group shortly behind middle of metatibial length, apical group at three quarters of metatibial length; basally with a few strong short single setae in coarse puncture with serrated borders; lateral face moderately densely and coarsely punctate, shortly setose; ventral edge finely serrated, with four robust equidistant setae; medial face impunctate; apex weakly truncate interiorly near tarsal articulation. Tarsomeres ventrally with sparse, short setae; not carinate laterally, impunctate dorsally; metatarsomeres with a strongly serrated ventral ridge; first metatarsomere distinctly shorter than following two tarsomeres combined and slightly longer than dorsal tibial spur. Protibia moderately long, bidentate; anterior claws symmetrical, basal tooth of inner claw sharply truncate at apex.

Aedeagus: Fig. 4I–K.

#### Diagnosis.

*Neoserica curvipenis* sp. n. differs from the two previous species by the extremely widely curved right paramere exceeding significantly beyond the level of the left paramere (in dorsal view).

#### Etymology.

The name of the new species is derived from the combined Latin words, *curvi* – curved, and *penis* – aedeagus, with reference to the curved shape of the right paramere.

#### Variation.

Body length: 6.6–8.1 mm, length of elytra: 4.9–5.6 mm, width: 4.0–4.4 mm. Female: antennal club composed of four antennomeres, as long as the remaining antennomeres combined.

**Figure 5.**
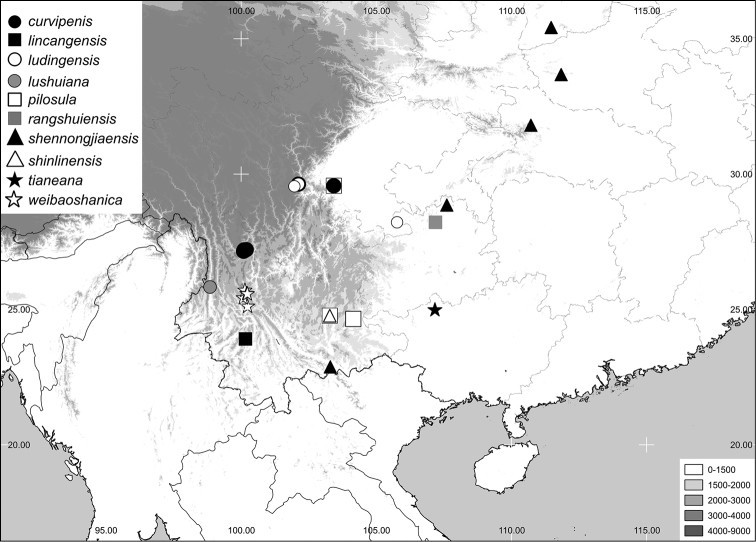
Distribution of the species of the *Neoserica pilosula* group.

## Supplementary Material

XML Treatment for Neoserica (s.l.) pilosula

XML Treatment for Neoserica (s.l.) ludingensis

XML Treatment for Neoserica (s.l.) weibaoshanica

XML Treatment for Neoserica (s.l.) tianeana

XML Treatment for Neoserica (s.l.) shennongjiaensis

XML Treatment for Neoserica (s.l.) lincangensis

XML Treatment for Neoserica (s.l.) rangshuiensis

XML Treatment for Neoserica (s.l.) lushuiana

XML Treatment for Neoserica (s.l.) emeishanensis

XML Treatment for Neoserica (s.l.) curvipenis

## References

[B1] Ahrens D (2003) Zur Identität der Gattung *Neoserica* Brenske, 1894, nebst Beschreibung neuer Arten (Coleoptera, Melolonthidae, Sericini). Koleopterologische Rundschau 73: 16–226

[B2] Ahrens D (2004) Monographie der Sericini des Himalaya (Coleoptera, Scarabaeidae). Dissertation. de - Verlag im Internet GmbH, Berlin, 534 pp

[B3] Ahrens D Liu WG Fabrizi S Bai M Yang XK (2014a) A taxonomic review of the *Neoserica* (sensu lato) *septemlamellata* group (Coleoptera: Scarabaeidae: Sericini). Zookeys 402: 76–102. doi: 10.3897/zookeys.402.7360 10.3897/zookeys.402.7360 PMC4023254 24843263

[B4] Ahrens D Liu WG Fabrizi S Bai M Yang XK (2014b) A taxonomic review of the *Neoserica* (sensu lato) *abnormis* group (Coleoptera: Scarabaeidae: Sericini). ZooKeys 402: 67–102. doi: 10.3897/zookeys.402.7360 25317056 10.3897/zookeys.439.8055 PMC4196255

[B5] Ahrens D Liu WG Fabrizi S Bai M Yang XK (in press) A revision of the species of the *Neoserica* (sensu lato) *vulpes* group (Coleoptera: Scarabaeidae: Sericini). Journal of Natural History.

[B6] Ahrens D Vogler AP (2008) Towards the phylogeny of chafers (Sericini): analysis of alignment-variable sequences and the evolution of segment numbers in the antennal club. Molecular Phylogenetics and Evolution 47: 783–798. doi: 10.1016/j.ympev.2008.02.010 18372194 10.1016/j.ympev.2008.02.010

[B7] Moser J (1915) Neue *Serica*-Arten. Deutsche Entomologische Zeitschrift 1915: 337–393

[B8] Pope RD (1960) *Aserica*, *Autoserica*, *Neoserica* or *Maladera*? (Col., Melolonthidae). Annals and Magazine of Natural History, Series 13, 3: 545–550

